# Supplementary material for: Development of Mia Phenotyping Using Paper-Based Device
Source: Diagnostics (Basel). 2022 Dec 9;12(12):3104. doi: 10.3390/diagnostics12123104 (PMC9777619; doi:10.3390/diagnostics12123104)

Figure S1. (A) Pixel intensity of different blood volumes (1, 2.5, and 5 µL) tested with paper-based device. RBC agglutination means positive result (left), while no RBC bound to antibody (non-agglutination) indicates negative result (right); (B) Reaction time between RBC and anti-Mi^{a}.

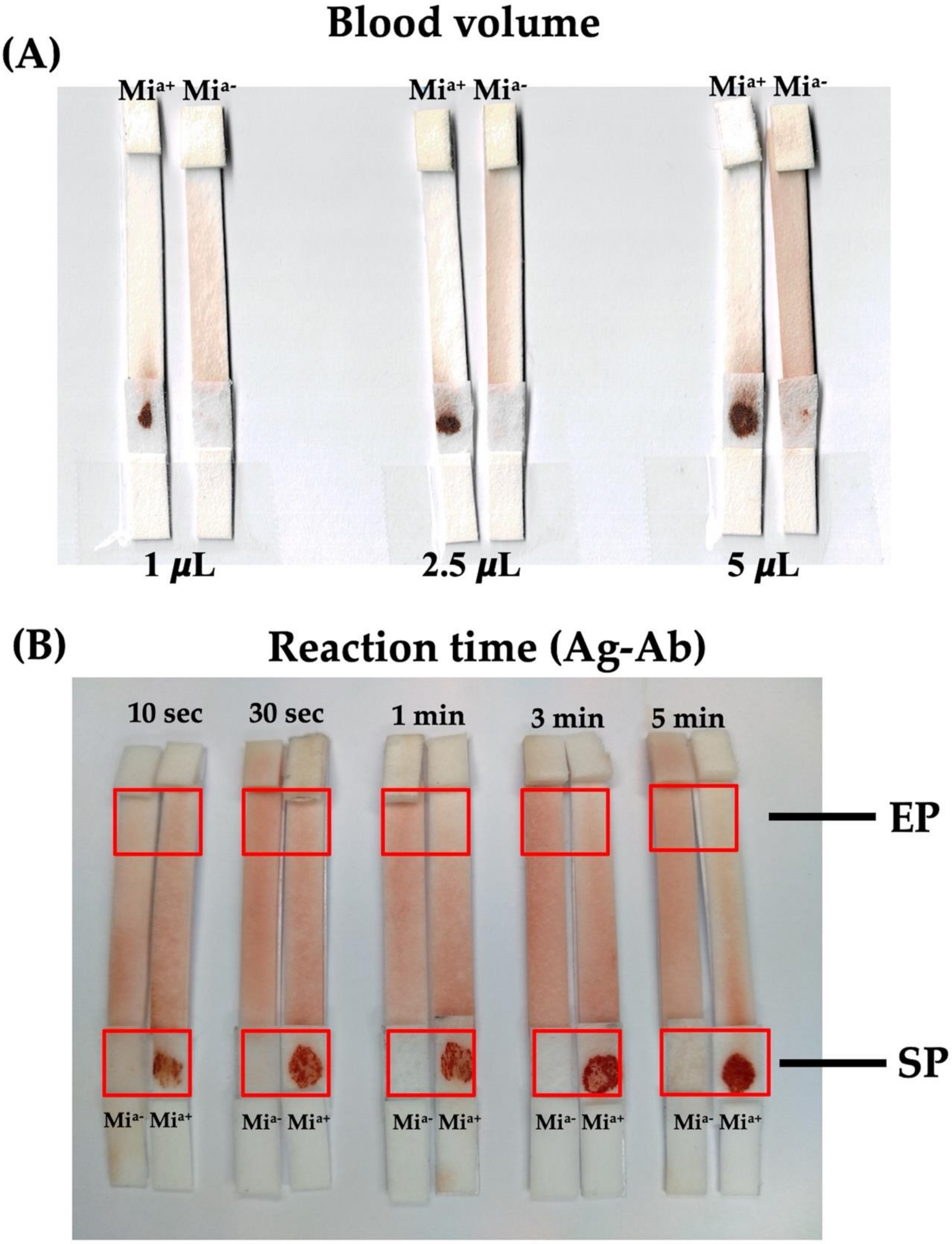

Figure S2. Mi^{a} blood typing results (samples 001–030).

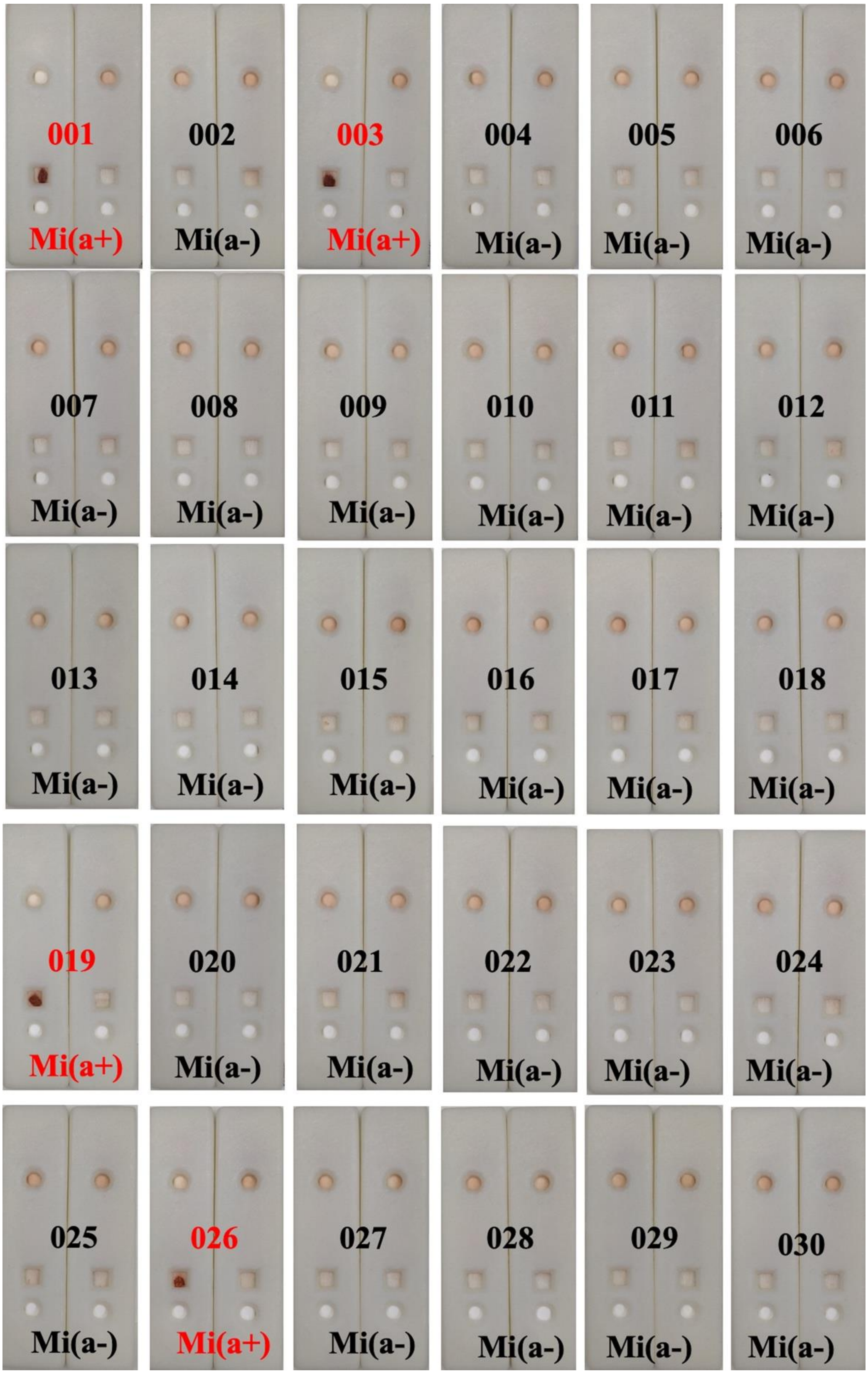

Figure S3. Mi^a blood typing results (samples 031–060).

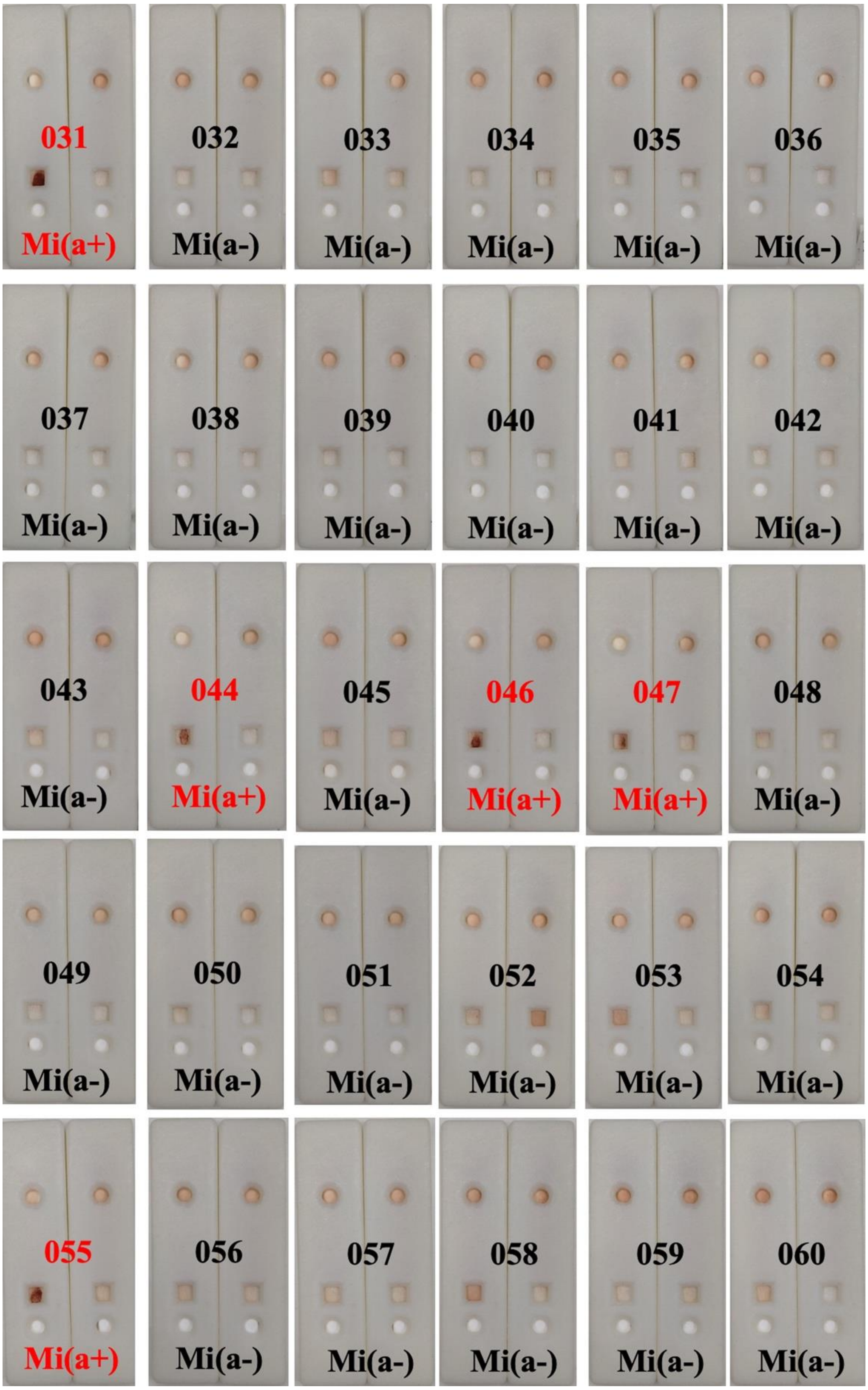

Figure S4. Mi^a blood typing results (samples 061–090).

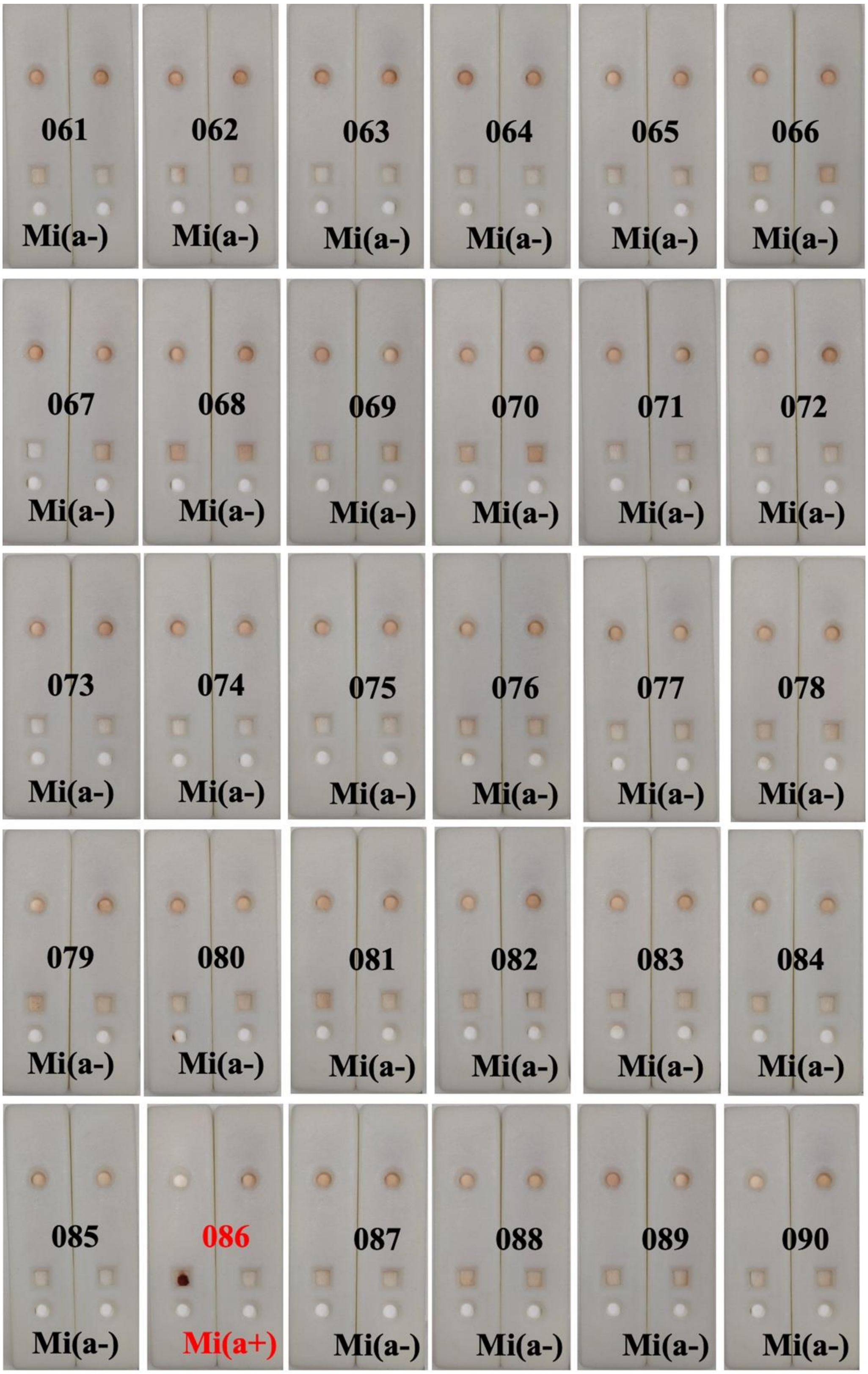

Figure S5. Mi^a blood typing results (samples 091–120).

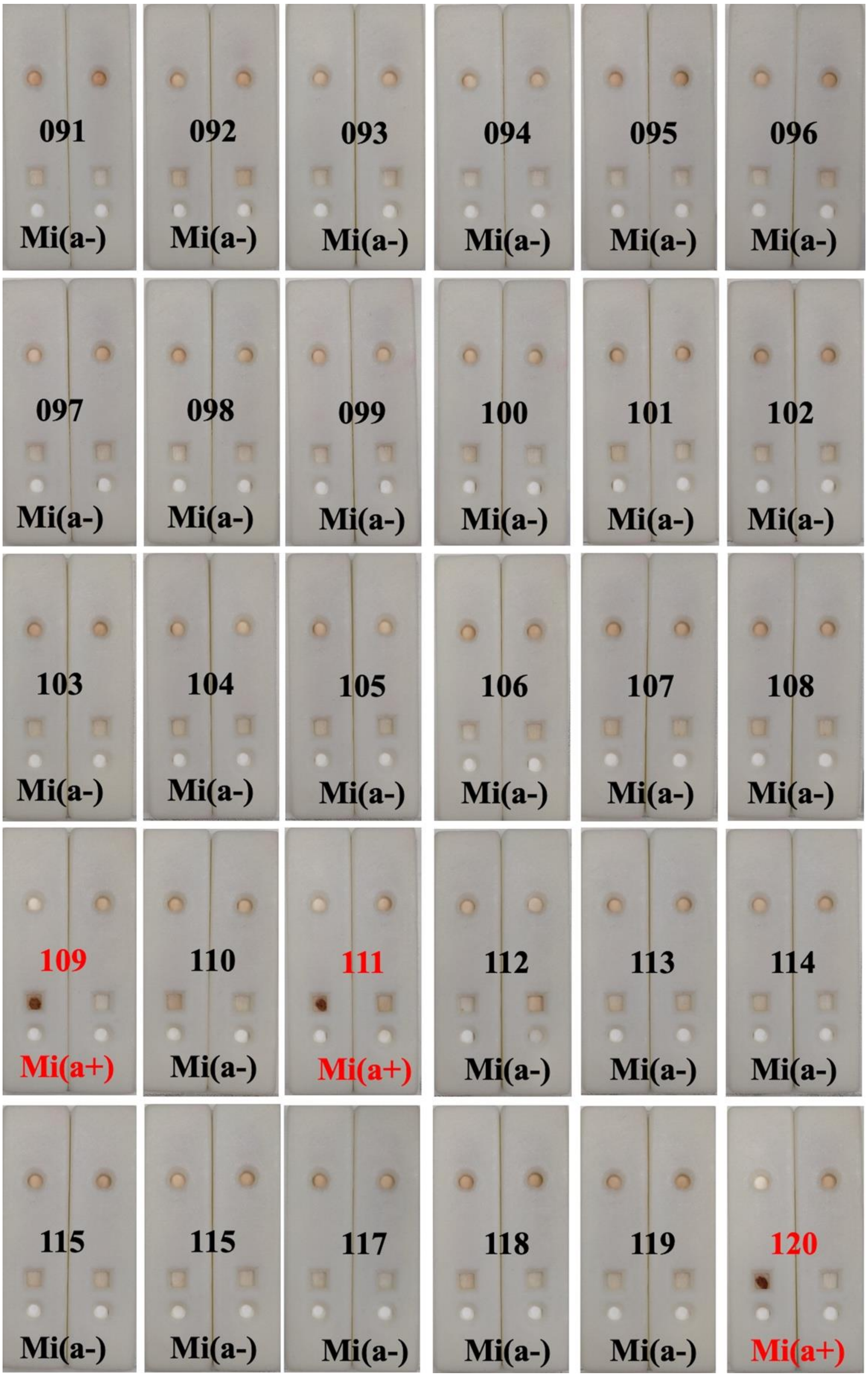

Figure S6. Mi^{a} blood typing results (samples 121–150).

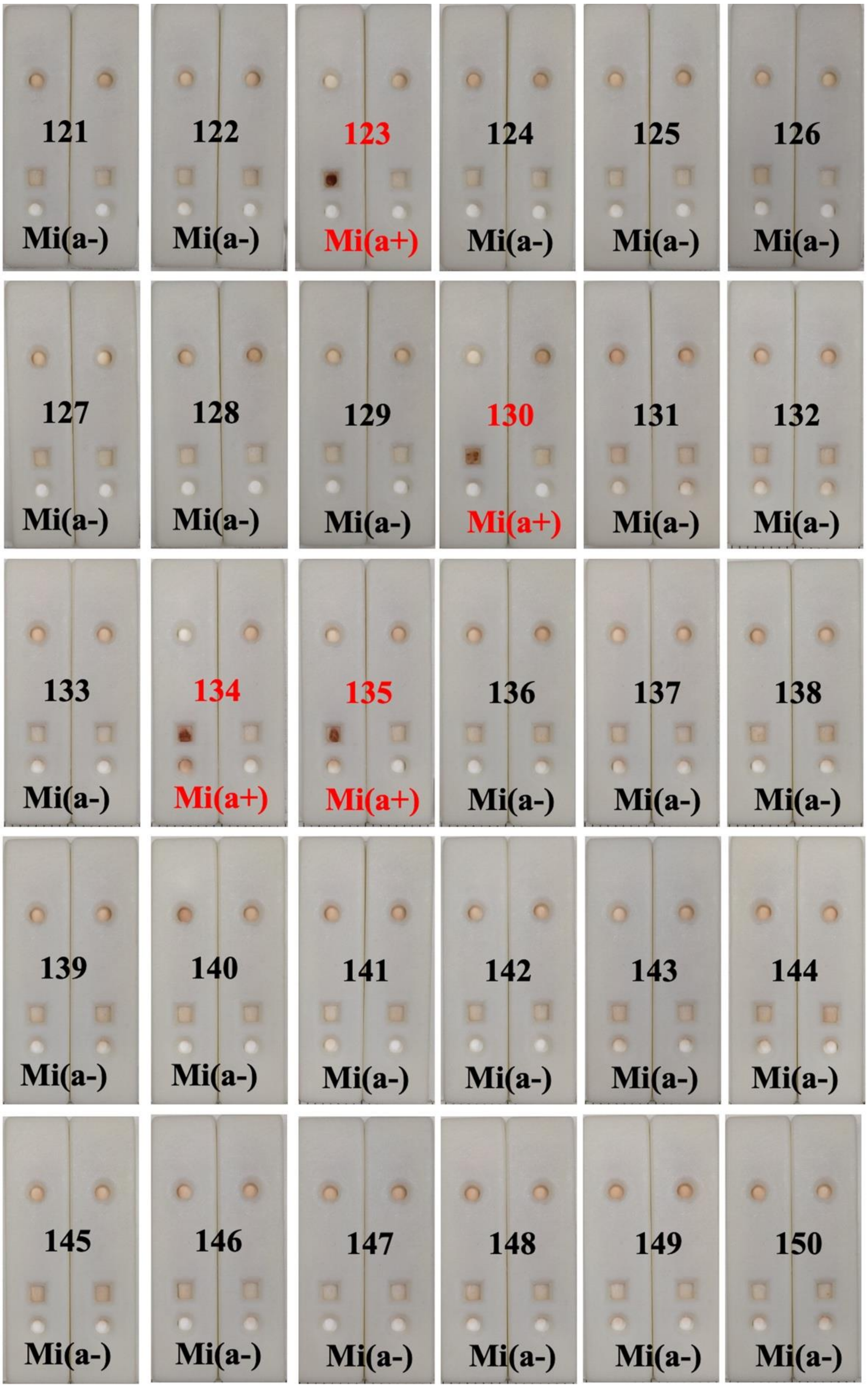

Figure S7. Mi^{a} blood typing results (samples 151–174).

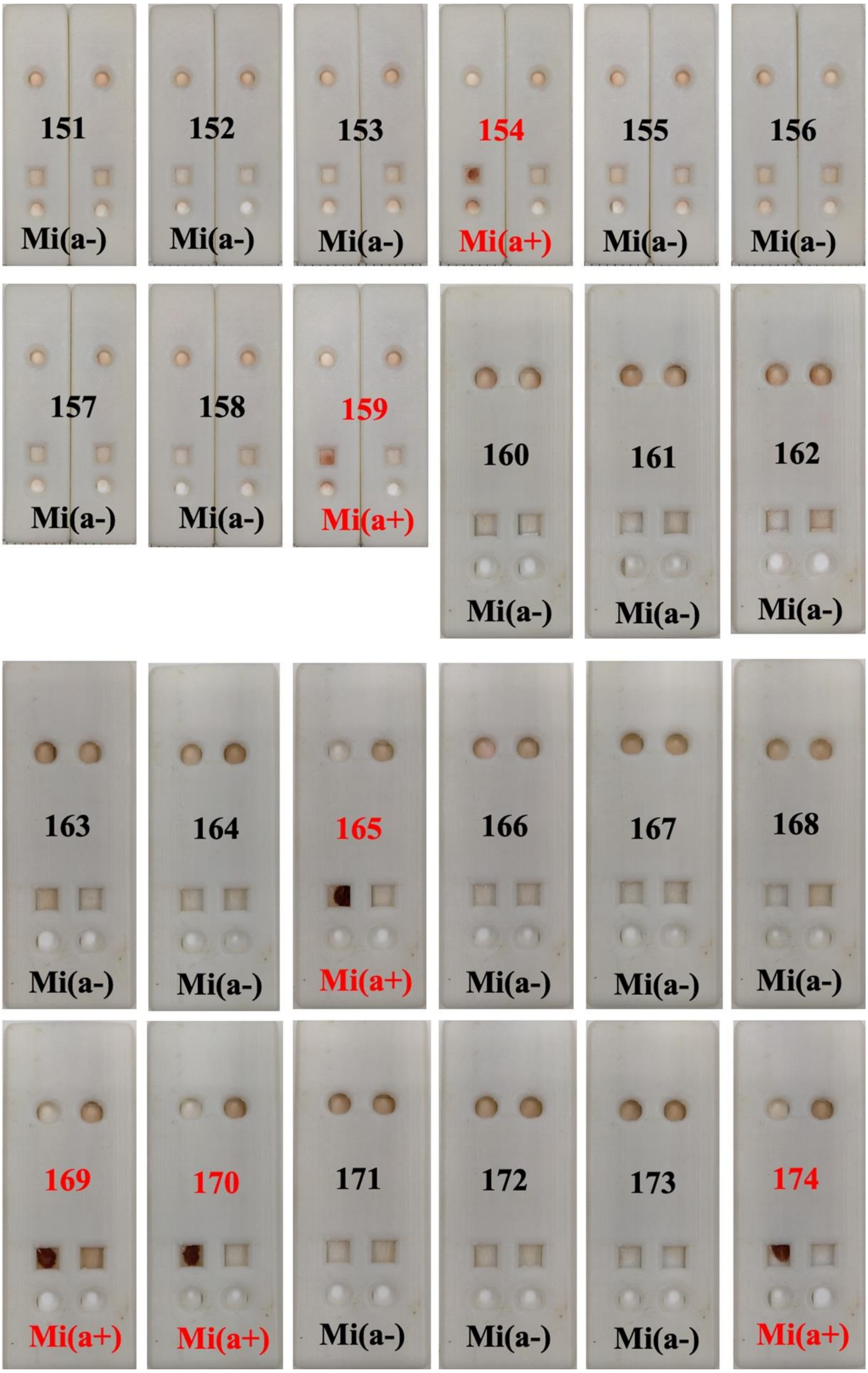

Figure S8. Mi^{a} blood typing results (samples 175–192).

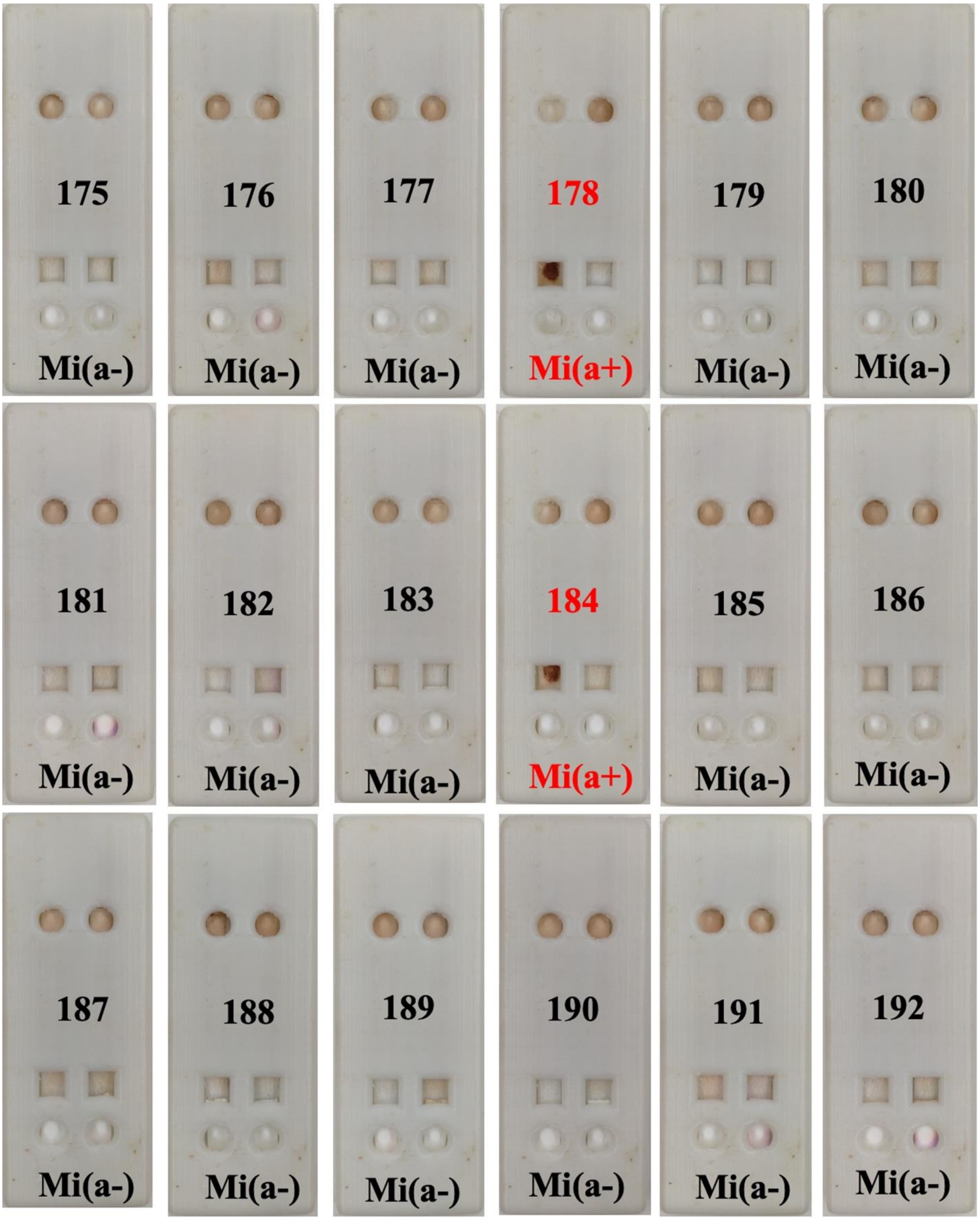

Figure S9. Mi^a blood typing results (samples 193–210).

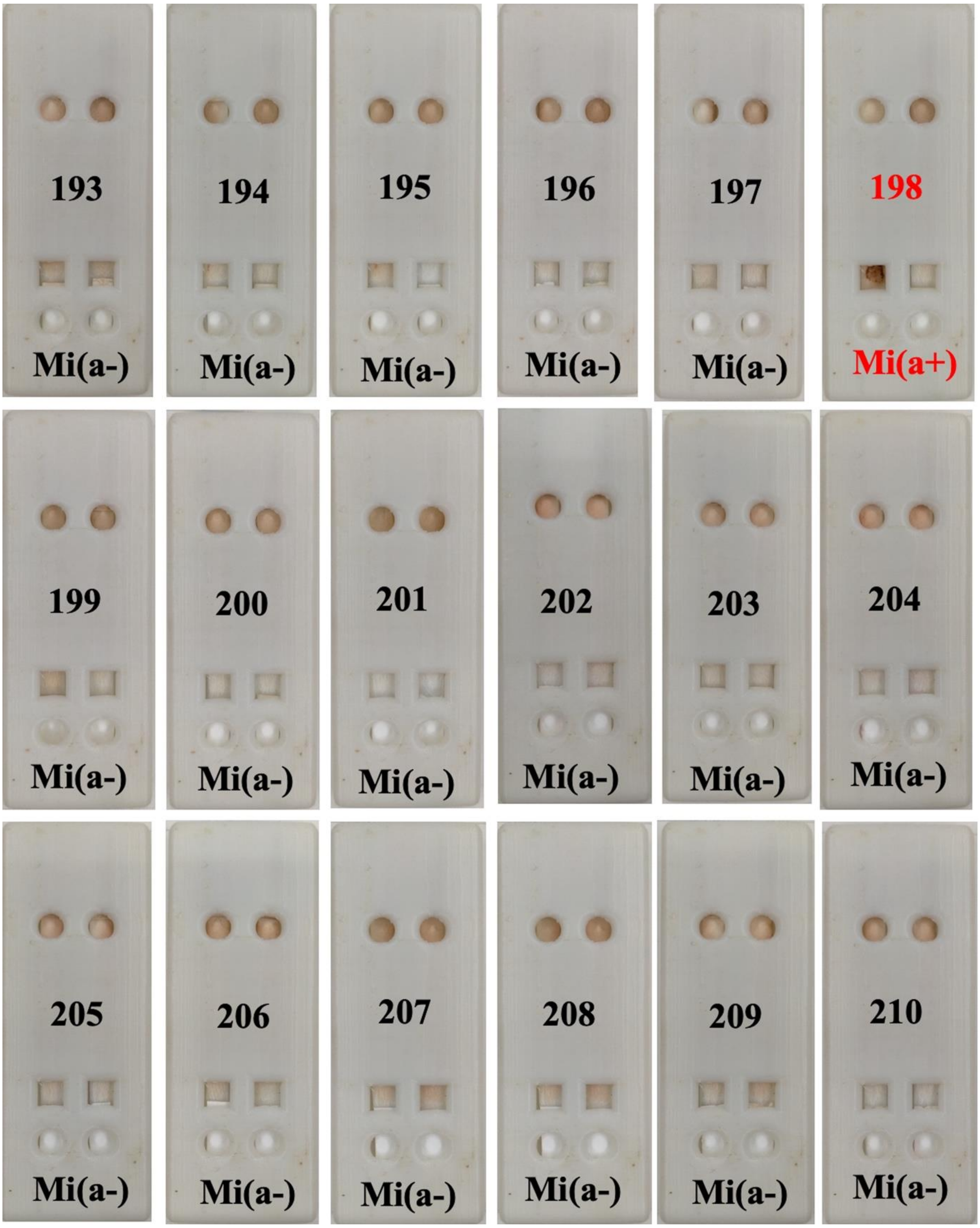

Figure S10. Mi^a blood typing results (samples 211–214).

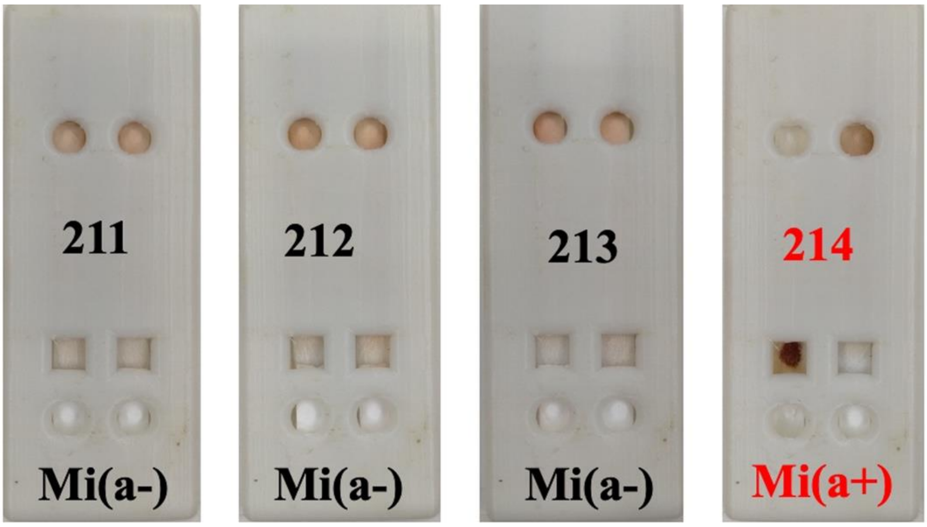

Supplement: Supplementary file 1 [file diagnostics-12-03104-s001.zip › diagnostics-2000692-supplementary.pdf]
